# Has China’s Healthcare Reform Reduced the Number of Patients in Large General Hospitals?

**DOI:** 10.3390/ijerph19095428

**Published:** 2022-04-29

**Authors:** Xiaojing Hu, Ping Wang

**Affiliations:** Medical Affairs Department, Peking University First Hospital, Beijing 100034, China; xiaojing.hu@pkufh.com

**Keywords:** healthcare reform, medical resource utilization, patients’ choice of hospital, large general hospital, healthcare system, utilization rate of outpatient appointments

## Abstract

Many studies have shown that the new round of healthcare reform launched by the Chinese government in 2009 has not effectively solved the problem in which patients more readily choose large general hospitals. We aimed to find out if this situation exists in every department of a large general hospital. This study collected the outpatient data of 24 departments for a large general hospital in Beijing. By calculating the average growth rate of outpatients in each department from 2014 to 2019, and the utilization rate of outpatient appointments in different departments in 2020, we found that the average growth rate of outpatients in 4 departments (16.6%) was negative, and the utilization rate of outpatient appointments in 13 departments (54.16%) was less than 80%. This shows that the number of patients in some departments is declining, and that there is an inefficient use of doctor resources. Obviously, this is inconsistent with people’s current beliefs. Therefore, it is not entirely true that China’s healthcare reform has not reduced the number of patients in large general hospitals. At the same time, the inefficient use of outpatient doctor resources is a phenomenon worthy of attention; if it persists, it will result in significant waste in the healthcare system. We suggest that policy makers and hospital managers in China, and countries similar to China, can attract attention and take measures.

## 1. Introduction

National health is an important livelihood-related issue that every government should pay attention to and continually optimize. As one of the many factors that can affect national health levels, the functioning of the healthcare system has always been a major concern. In order to better cope with the increasing healthcare challenges, and provide quality healthcare services, governments around the world have launched various forms of healthcare reform [1,2,3,4,5].

Although the policies and actions implemented differ between countries, in general, they are mainly focused on five aspects of improving the healthcare system, namely quality, accessibility, fairness, efficiency, and sustainability [6]. As a complex and significant systemic task, healthcare reform requires substantial and sustained investment in medical personnel, infrastructure, technology, drug supply, and quality-improvement systems. Even though policy makers have carefully designed healthcare reforms, their success ultimately depends on careful attention to complex details [7].

In March 2009, the Central Committee of the Communist Party of China and the State Council issued the “Opinions on Deepening Health System Reform” to launch a new round of healthcare reform [8]. The plan of this healthcare reform can be summarized as one goal, four beams, and eight columns. In order to achieve the goal of establishing a basic healthcare system to provide universal coverage, the Chinese government hopes to establish four systems, including a public health service system, a medical service system, a medical insurance system, and a drug supply and security system. The eight operating mechanisms (administration, operation, financing, pricing, governance, security for technology and human resources, information, and legislation) will provide necessary support for this [9]. The Chinese government has introduced several policies, such as the hierarchical diagnosis and treatment system [10,11,12], the family doctor signing system [13,14], the capacity improvement plan for primary medical institutions [15,16], universal health insurance [17], the comprehensive implementation of appointment registration [18,19,20], and various internet medical services [21,22,23]. The implementation of these policies has played a positive role in promoting the accessibility and fairness of medical services [24,25,26]. However, some studies have shown that the policies also present additional problems; that is, patients are diverted to large general hospitals instead of community hospitals as expected [27,28,29]. This is because when the total hospital cost is affordable, patients care more about medical services’ quality than the cost [30,31,32]. At the same time, due to the government’s low rate for medical services and hospitalization expenses [33], public hospitals, which should reflect more public welfare, often need to consider maximizing profits to subsidize employee wages [34]. Therefore, managers and doctors of large general hospitals have a strong motivation and willingness to provide more medical resources and services to attract more patients. The latest research results [35] are showing policy makers that their efforts have not worked, and that patients are still pouring into large general hospitals. However, is this true?

The aim of this study is to find that whether outpatient visits are continuing to increase in every department of large general hospitals and to determine what is the outpatient appointment rate that reflects the utilization of outpatient doctor resources in different departments.

## 2. Materials and Methods

### 2.1. Data Sources

Peking University First Hospital is a large general hospital ranked in the top 12 in China. More than 2 million patients use outpatient services at Peking University First Hospital every year. Outpatient data from a total of 24 departments were collected in this study. Each department’s outpatient data from 2014 to 2020 were retrospectively collected and sorted by one data collector, and another person checked them. The data came from the outpatient management information system of the Beijing Source Electronic Information Technology Company. No specific ethical approval was required, as no humans were involved in this study and no in vivo animal experiments were carried out. 

### 2.2. Definitions of Indicators

The outpatient in this study refers to the place where doctors conduct preliminary diagnosis of patients’ conditions in the hospital, in addition to providing medication prescriptions; it is also where hospitalization is conducted.

The average growth rate of outpatients in each department from 2014 to 2019 = [(N2019 − N2018)/N2018 + (N2018 − N2017)/N2017 + (N2017 − N2016)/N2016 + (N2016 − N2015)/N2015 + (N2015 − N2014)/N2014]/5 × 100%. N represents the number of outpatients in various years. As a result of the impact of the COVID-19 epidemic in 2020, the number of outpatients in global medical institutions declined significantly. In order to make the statistical data similar to actual, natural situations, we excluded the 2020 data when calculating the average growth rate of outpatients in various departments.

Utilization rate of outpatient appointment = (number of registered patients/total number of appointments available) × 100%; the number of registered patients is the number of successful registration of outpatients in various departments through various appointment channels during the investigation period; the total number of appointments available is the total number provided by each department through all appointment channels during the investigation period.

### 2.3. Data Analysis

SPSS 24.0 was used for descriptive statistical analysis of the ratio-based data of the average growth rate of outpatients and the utilization rate of outpatient appointments; the differences between the two indicators in 24 departments were compared.

## 3. Results

### 3.1. Average Growth Rates of Outpatient Visits in Each Department from 2014 to 2019

In all 24 departments, there were six departments (25.00%) whose annual year-on-year growth rate was less than zero for three time periods; there were eight departments (33.33%) whose annual year-on-year growth rate was less than zero for two time periods; and there were four departments (16.67%), including pediatrics, orthopedics, neurology, and integrated traditional Chinese and Western medicine, in which the average growth rate of outpatient visits from 2014 to 2019 was less than zero, as shown in Table 1.

### 3.2. Utilization Rate of Outpatient Appointments in Each Department

More than 50% (13/24) of the departments’ outpatient appointment utilization rates were less than 80% in all departments. Departments such as general practice, integrated traditional Chinese and Western medicine, radiotherapy, and cancer chemotherapy had less than a 60% utilization rate for outpatient appointments. A total of 11 departments (45.83%) had a utilization rate for outpatient appointments exceeding 80%, among which three departments (12.5%)—dermatology and venereal diseases, cardiovascular medicine, and digestive medicine—exceeded 100% (see Table 2).

### 3.3. The Average Outpatient Growth Rate—Utilization Rate of Outpatient Appointments in Different Departments

**Figure 1:**
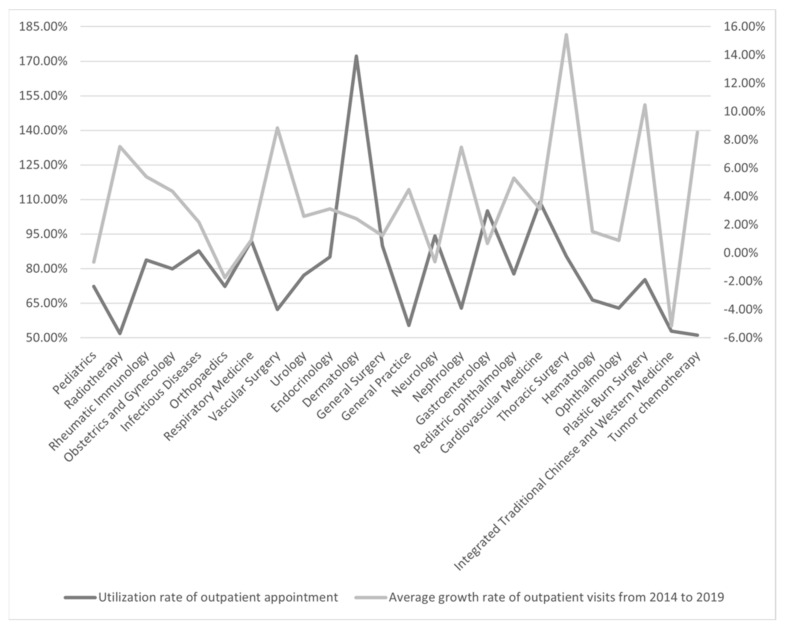
The average outpatient growth rate—utilization rate of outpatient appointments in different departments.

As Figure 1 shows, the average outpatient growth rate in three departments is negative, and the utilization rate for outpatient appointments is less than 80%. There are ten departments where the average outpatient growth rate is positive but the utilization rate for outpatient appointments is less than 80%.

## 4. Discussion

In this study, we took Peking University First Hospital, which is in the top 12 of China’s high-level large general hospitals, as a sample. By analyzing the change in outpatient numbers in the hospital’s different departments from 2014 to 2019, we calculated the actual utilization rate of outpatient appointments in different departments in 2020. We found some interesting and noteworthy phenomena that differ from current research results. We believe that our results will provide policy makers and hospital managers some new inspiration and thinking.

Contrary to previous research, our study found at present that in some departments of Peking University First Hospital, which represents the highest medical level in China, there has been a decline in the number of patients. This is the expected result of healthcare reform. In order to explain this phenomenon, it is necessary to first know that there has been no specific general physician GP or a clear referral system in China’s healthcare system for a long time. Therefore, when an individual is ill, only the patient decides which level of medical institution to choose. Therefore, changes in the number of outpatient visits in a hospital and in one of its departments depend on the willingness of patients to choose it. Previous studies have shown that the influencing factors of patients’ willingness to choose medical institutions at different levels include personal preferences (mainly for hospital/doctor visibility), disease type and severity, economic capacity, medical technology, medical insurance, culture and customs, and the distance between medical institutions and patients’ homes [36,37,38,39,40,41]. Among them, medical technology, the distance between medical institutions and patients’ homes, economic factors, and the severity of diseases are considered to be the most important factors affecting patients’ choice of healthcare providers [42,43]. In addition, some studies have shown that the accurate transmission of information on healthcare reform to patients can also help to improve patients’ satisfaction with reform and low-level hospitals, and thus encourage patients to prioritize choosing low-level hospitals [44].

We believe that it is precisely because the new round of healthcare reforms has affected these factors that the current results have been achieved. For example, the country established a sound hierarchical medical system and an online appointment system. In the report on the two sessions of the government work of China in 2021, it was once again explicitly proposed to accelerate the construction of a hierarchical medical system [45]. The “Notice on deepening the convenience and benefit activities of Internet + medical health” released in 2018 also stated that all regions should establish and improve online appointment service platforms and prioritize reserving appointment sources for primary medical and health institutions in the medical alliance [20]. These two policies effectively promoted the implementation of primary referral and smooth two-way referral. Studies have shown that if the medical system allows referrals and online appointments to occur at the same time, the distribution of patients at different levels of medical institutions becomes the most reasonable [46]. Especially for urban residents, hierarchical diagnosis and treatment policy have a significant impact on patients’ healthcare-seeking habits [11]. Therefore, China’s current hierarchical medical system and online appointment system will become an effective policy tool for patients to choose hospitals, and realize the rational allocation of existing medical resources [46].

Another important policy is related to the family doctor contract system. In 2016, the government issued the “Notice on the issuance of promotion guidelines for family practice signing services” [13]; it proposed actively piloting the family doctor contract service and then promoting the family doctor contract service nationwide by 2020. A number of studies have shown that contracts for family doctors have played a positive role in the management of patients with various chronic diseases [47,48,49]. An increasing number of patients with chronic diseases benefit from family doctors, meaning that they do not have to go to the hospital for routine drugs and basic examinations.

At the same time, the medical insurance payment policy has also made several efforts in guiding patients to primary medical institutions, including the introduction of gradient reimbursement plans [50]. In most regions, the reimbursement gap between different levels of medical institutions is maintained at 5–10%. Reimbursement for patients directly to high-level hospitals without a referral will be reduced by 10–20% or may not be covered by medical insurance, as a result of non-compliance with reimbursement conditions [51]; this plays a vital role in regulating patients’ healthcare-seeking behavior.

In addition, in recent years the health committee has been promoting the capacity improvement plan for primary medical institutions to improve the service capacity of primary medical institutions continuously; this also appears in this year’s governmental work report [45]. With the improvement in the ability of primary medical institutions to diagnose and treat conditions, many diseases that were previously untreatable at primary medical institutions can now be better treated. Therefore, patients have no incentive to spend more time and financial costs (such as medical and travel costs) to go to remote large general hospitals. Patients’ healthcare-seeking behavior has also become more rational. In the past, regardless of whether they were experiencing a major or minor illness, they chose to go to large general hospitals; in contrast, they are now choosing to go to large general hospitals only if they are suffering from complex miscellaneous diseases that cannot be diagnosed or treated in local hospitals.

Finally, in the past five years, in order to implement various policies, the central government has included relevant indicators in the performance evaluation of local governments and medical institutions at all levels. Therefore, local governments and medical institutions at all levels have paid more attention to various healthcare reform policies, and the intensity of implementation and publicity has become stronger. This is especially notable considering that, in China, higher-level governments have the right to promote local officials; this motivates local government officials at all levels to resolutely implement national policies, since it will make it easier for them to obtain formal or informal recognition from higher-level policy supporters and thereby promote the development of personal undertakings [52]. Obviously, the attention of local governments helps to accurately transmit healthcare reform information to patients.

However, our study shows that only some departments in large general hospitals have experienced a decline in outpatient visits, which is mainly a result of patients’ preferences. A distinct feature of China’s large general hospitals is that each hospital has its advantages in various departments and top experts, which makes it difficult to achieve top-level positions in all departments. Therefore, when patients demand medical treatment in a particular department, priority will be given to choosing a specific large general hospital with advantages in the relevant department, even if the doctor resources in that department are significantly constrained. Patients will also choose to use various social relations, and even purchase doctor resources through informal channels such as “huangniu”, rather than go to other large general hospitals. This may also indicate that the current policies of healthcare reform make patients no longer blindly pursue the reputation of large general hospitals when they need to see a doctor. However, for departments and well-known experts with technical advantages in large general hospitals, the effect that policies of healthcare reform have on patients’ preferences should not be temporary. This could be a future focus for policy makers.

The fact that our findings are inconsistent with the results of the existing literature may have the following explanations. Firstly, any policy that aims to produce the expected effect will go through a period of time lag. Only after fully understanding and evaluating the policy’s advantages and disadvantages can people act as envisioned by policy makers. Secondly, for such complex problems as patients’ preferences for large general hospitals, multiple policies are often needed to play a comprehensive role, and the promulgation of policies is often gradual and does not occur overnight. However, many previous studies may not accurately represent the most recent situation because the data used are not the latest. In addition, although there are large differences between medical institutions in China, most of the data for research on such issues are integrated with the average values of various types of hospital data in China. On average, patients prefer large general hospitals when seeking medical treatment compared with primary medical institutions, but this conclusion may not be popular in certain types of hospitals and departments.

In addition, we also found there has been inefficient use of outpatient doctor resources in some departments. According to prior literature [53] and our experience, the sufficient utilization rate of outpatient appointments in departments is within the range of 80–100%, which can best meet the needs of patients, and will not cause obvious waste of resources. Therefore, in this study, we use 80% as the cut-off point to assess whether there is insufficient use of outpatient doctor resources in a department. We found that out of 24 departments, 13 had insufficient utilization of outpatient doctor resources. Combined with the growth rate of outpatient visits, it can be found that among the four departments with decreased outpatient visits, three departments have insufficient use of outpatient doctor resources.

This phenomenon should undoubtedly be of concern. If this inefficient use is not dealt with effectively, it may result in a serious waste of resources. Previous studies [54,55] have shown that, when medical insurance coverage is universal, there is a moral hazard—medical service providers may over-provide medical services, and patients may overuse medical services. For example, expensive tests such as transthoracic echocardiography [56] and various imaging examinations [57,58,59] are performed in patients with low probability of pre-examination. This moral hazard and the service fee system are considered the main factors responsible for the oversupply of medical services and the resulting medical waste in China; the excessive waste of resources, in turn, further hinders the process of realizing universal medical coverage [34]. This situation occurs despite sufficient sources of patients. Once sources of patients become insufficient, we believe that the possibility of excessive provision of medical services by providers will increase significantly, causing more serious waste of medical resources and increased medical costs.

Whether outpatient doctor resources are fully utilized depends on the supply of doctors and the number of patients. For departments where the number of patients decreases and outpatient doctor resources are underutilized, we believe that the inefficient use of outpatient doctor resources is mainly due to the fact that most hospitals in China still rely on personal experience to estimate the demand for doctors in each visiting unit. However, the extensive outpatient plan lacks accuracy and dynamics. As a result, when various factors result in a decrease in the demand for patients in some departments, if there is no timely and effective adjustment, it results in a decrease in the effective utilization of doctors’ resources and an increase in unnecessary medical service costs. Nevertheless, the manual outpatient plan cannot ensure the lowest total cost for human resources, or fully reflect the humanization and fairness of outpatient management; this is also not conducive to improving the enthusiasm of doctors [60].

For departments where the number of patients remains stable or increases, there is still an insufficient use of outpatient doctor resources. We believe a reasonable explanation for this is that managers in large general hospitals have become accustomed to providing as much medical resources as possible without realizing the potential waste of resources. These resources beyond demand can be allocated to low-level medical institutions for technical assistance, for training doctors in low-level medical institutions, or remote consultation using digital technology; we believe these adjustments will help to further reduce the reduction in patients in large general hospitals and make full use of resources. Of course, there will be some difficulties, such as doctors’ personal motivations, the differences between different disciplines, the inefficient use of doctors’ time costs, overhead costs, and staffing costs, resulting in increased overall healthcare costs [61,62]. Therefore, determining how to make full use of doctor resources in large general hospitals to help low-level medical institutions may also become the focus of future healthcare reform policies.

Of course, our research also has some shortcomings. First of all, we need to study more hospitals at the same level as the First Hospital of Peking University to determine whether the current decline in the number of patients in some departments and the insufficient use of doctors are a special case, or common in more high-level large general hospitals in China. Secondly, in future research, we can further use the method of health economics to quantify the waste caused by the inefficient use of doctor resources. Finally, if we can obtain data via qualitative interviews with patients, this would be useful to ascertain reasons for why patients choose smaller or larger institutional health services, which would supplement our own findings as well.

## 5. Conclusions

China’s healthcare reform has not completely solved the problem of having too many patients seeking out large general hospitals for care and treatment. However, healthcare reform policy has produced some positive effects. We have seen that the number of patients in some departments is declining. Of course, we should also pay attention to the accompanying insufficient use of outpatient doctor resources.

Therefore, we suggest that policy makers and hospital managers should pay attention not only to the overall situation represented by averages, but also to the personalization represented by some typical hospitals. If we can reduce patients’ preferences for choosing famous departments and doctors in large general hospitals, and find ways to make better use of doctors’ resources in large general hospitals to help low-level medical institutions, this may help to better allocate medical resources at all levels, provide full use for their value, and prevent waste.

## Figures and Tables

**Table 1:** Average growth rates of outpatient visits in each department from 2014 to 2019.

Departments	Growth Rate, 2014–2015	Growth Rate, 2015–2016	Growth Rate, 2016–2017	Growth Rate, 2017–2018	Growth Rate, 2018–2019	Growth Rate, 2014–2019
Pediatrics	−2.82%	5.26%	−5.97%	−4.53%	4.79%	−0.65%
Radiotherapy	3.23%	8.62%	3.17%	3.32%	19.21%	7.51%
Rheumatic Immunology	3.19%	14.41%	−6.63%	7.05%	8.89%	5.38%
Obstetrics and Gynecology	5.12%	12.69%	−13.09%	3.33%	13.75%	4.36%
Infectious Diseases	8.27%	−2.11%	−5.66%	3.37%	7.01%	2.18%
Orthopedics	1.27%	0.72%	−12.73%	−4.47%	6.46%	−1.75%
Respiratory Medicine	−1.56%	7.21%	−4.11%	−1.65%	4.72%	0.92%
Vascular Surgery	6.43%	14.81%	1.58%	11.12%	10.26%	8.84%
Urology	5.60%	−2.29%	−3.66%	6.16%	7.10%	2.58%
Endocrinology	5.73%	8.90%	−9.99%	1.22%	9.73%	3.12%
Dermatology	3.59%	6.35%	−5.71%	−0.62%	8.47%	2.42%
General Surgery	7.12%	−2.07%	−4.64%	−1.19%	6.88%	1.22%
General Practice	−8.40%	5.30%	5.39%	17.16%	2.96%	4.48%
Neurology	−1.17%	−1.39%	−7.12%	0.58%	5.94%	−0.63%
Nephrology	−2.23%	15.91%	−0.10%	14.96%	8.88%	7.48%
Gastroenterology	3.52%	−0.11%	−12.15%	10.07%	2.09%	0.68%
Pediatric ophthalmology	−6.66%	10.47%	7.52%	1.03%	14.13%	5.30%
Cardiovascular Medicine	8.30%	0.60%	−16.00%	8.79%	13.90%	3.12%
Thoracic Surgery	14.87%	16.42%	8.19%	12.53%	25.15%	15.43%
Hematology	3.68%	5.32%	−8.36%	−1.10%	8.06%	1.52%
Ophthalmology	2.92%	5.38%	−6.29%	−0.93%	3.34%	0.89%
Plastic Burn Surgery	22.39%	4.54%	3.49%	−0.26%	22.14%	10.46%
Integrated Traditional Chinese and Western Medicine	−5.69%	1.71%	−17.57%	−4.79%	0.50%	−5.17%
Tumor chemotherapy	−2.73%	5.95%	−11.13%	−10.73%	61.28%	8.53%

**Table 2:** Utilization rates of outpatient appointments in each department.

Departments	Total Number of Appointments Available	Number of Registered Patients	Utilization Rate
Dermatology	53,192	91,614	172.23%
Cardiovascular Medicine	56,833	61,840	108.81%
Gastroenterology	49,731	52,257	105.08%
Neurology	52,187	49,141	94.16%
Respiratory Medicine	34,714	31,979	92.12%
General Surgery	41,767	37,562	89.93%
Infectious Diseases	19,758	17,338	87.75%
Thoracic Surgery	12,777	10,902	85.33%
Endocrinology	88,898	75,568	85.01%
Rheumatic Immunology	34,429	28,816	83.70%
Obstetrics and Gynecology	222,569	178,061	80.00%
Pediatric ophthalmology	29,921	23,268	77.76%
Urology	125,285	96,556	77.07%
Plastic Burn Surgery	7410	5573	75.21%
Orthopedics	52,895	38,263	72.34%
Pediatrics	58,617	42,350	72.25%
Hematology	19,555	12,999	66.47%
Nephrology	97,849	61,534	62.89%
Ophthalmology Department	60,421	37,961	62.83%
Vascular Surgery	18,379	11,445	62.27%
General Practice	8387	4643	55.36%
Integrated Traditional Chinese and Western Medicine	76,203	40,273	52.85%
Radiotherapy	8321	4312	51.82%
Tumor chemotherapy	9110	4652	51.06%

Note: In some departments, the utilization rate of outpatient appointments exceeded 100%, since the number of registered persons was more than the total number of appointment sources actually provided by the department; doctors provide outpatient services for the extra patients via overtime.

## Data Availability

Not applicable.

## References

[1] Friebel R., Molloy A., Leatherman S., Dixon J., Bauhoff S., Chalkidou K. (2018). Achieving high-quality universal health coverage: A perspective from the National health service in England. BMJ Glob. Health.

[2] Clemens T., Michelsen K., Commers M., Garel P., Dowdeswell B., Brand H. (2014). European hospital reforms in times of crisis: Aligning cost containment needs with plans for structural redesign?. Health Policy.

[3] Marten R., McIntyre D., Travassos C., Shishkin S., Longde W., Reddy S., Vega J. (2014). An assessment of progress towards universal health coverage in Brazil, Russia, India, China, and South Africa (BRICS). Lancet.

[4] Almeida G., Sarti F.M. (2013). Measuring evolution of income-related inequalities in health and health care utilization in selected Latin American and Caribbean countries. Rev. Panam. Salud Pública.

[5] Lagomarsino G., Garabrant A., Adyas A., Muga R., Otoo N. (2012). Moving towards universal health coverage: Health insurance reforms in nine developing countries in Africa and Asia. Lancet.

[6] Pőlluste K., Kallikorm R., Meiesaar K., Lember M. (2012). Satisfaction with access to health services: The perspective of Estonian patients with rheumatoid arthritis. Sci. World J..

[7] Comber A.J., Brunsdon C., Radburn R. (2011). A spatial analysis of variations in health access: Linking geography, socio-economic status and access perceptions. Int. J. Health Geogr..

[8] National Development and Reform Commission of People’s Republic of China (2009). Opinions of the CPC Central Committee and the State Council on Deepening the Health Care System Reform. http://www.china.org.cn/government/scio-press-conferences/2009-04/09/content_17575378.html.

[9] Tao W., Zeng Z., Dang H., Lu B., Chuong L., Yue D., Wen J., Zhao R., Li W., Kominski G.F. (2020). Towards universal health coverage: Lessons from 10 years of healthcare reform in China. BMJ Glob. Health.

[10] Meng Q.Y., Mills A., Wang L.D., Han Q.D. (2019). What can we learn from China’s health system reform?. BMJ.

[11] Zhou Z.L., Zhao Y.X., Shen C., Lai S., Nawaz R., Gao J.M. (2021). Evaluating the effect of hierarchical medical system on health seeking behavior: A difference-in-differences analysis in China. Soc. Sci. Med..

[12] Lian L., Zou M., Wang X., Chen J. (2019). Building the tiered system of disease diagnosis and treatment from 2015 to 2017 in Jiangsu: Achievements and challenges. Lancet.

[13] State Council Medical Reform Office, National Health and Family Planning Commission, National Development and Reform Commission, Ministry of Civil Affairs, Ministry of Finance, Ministry of Human Resources and Social Security and State Administration of Traditional Chinese Medicine (2016). Guidance on the Promotion of Family Practice Contract Service. http://www.gov.cn/xinwen/2016-06/06/content_5079984.htm.

[14] National Health Commission, State Administration of Traditional Chinese Medicine (2018). Guidance on Regulating the Management of Family Doctor Signing Service. http://www.gov.cn/gongbao/content/2019/content_5363082.htm.

[15] National Health and Family Planning Commission, State Administration of Traditional Chinese Medicine (2017). Implementation Plan for Annual Activities to Improve Primary Health Service Capacity. http://www.nhc.gov.cn/jws/s3581/201704/577dbceb161e480589173ef87b1b7721.shtml.

[16] National Health Commission (2020). Notice on Comprehensively Promoting Community Hospital Construction. http://www.nhc.gov.cn/jws/s3581/202007/2aab83700656411e9ab35ae9049dc732.shtml.

[17] Frazier M.W. (2014). State schemes or safety nets? China’s push for universal coverage. Daedalus.

[18] National Health and Family Planning Commission (2009). Opinions on Implementing Appointment Service in Public Hospitals. http://www.nhc.gov.cn/bgt/s9514/200909/fe1e60fab1a0456cae8a70cadd42ef57.shtml.

[19] National Health and Family Planning Commission (2011). Notice on Further Promoting Appointment Medical Service. http://www.nhc.gov.cn/yzygj/s3573/201307/cac78732de154b1b8db8806913ffb6e2.shtml.

[20] National Health Commission (2020). Notice on Further Perfecting Appointment System and Strengthening the Construction of Smart Hospital. http://www.gov.cn/zhengce/zhengceku/2020-05/22/content_5513897.htm.

[21] State Council (2018). Opinions on Promoting the Development of ‘Internet + Medical Health’. http://www.gov.cn/zhengce/content/2018-04/28/content_5286645.htm.

[22] National Health Commission, State Administration of Traditional Chinese Medicine (2018). Notice on Deepening the Convenience and Benefit Activities of ‘Internet + Medical Health’. http://gcs.satcm.gov.cn/zhengcewenjian/2018-07-18/7410.html.

[23] National Health Commission, State Administration of Traditional Chinese Medicine (2018). Internet Diagnosis and Treatment Management Measures (Trial), Internet Hospital Management Measures (Trial), Remote Medical Service Management Specification (Trial). http://bgs.satcm.gov.cn/zhengcewenjian/2018-09-17/7909.html.

[24] Lv H., Gu J., Yuan X., Miao Y. (2020). Prioritizing the perceived equity of the residents to construct an equitable health care system: Evidence from a national cross-sectional study in China. BMC Health Serv. Res..

[25] Tao W., Zeng W., Yan L., Yang H., Wen J., Li W. (2019). The health service capacity of primary health care in West China: Different perspectives of physicians and their patients. BMC Health Serv. Res..

[26] Yuan B., Balabanova D., Gao J., Tang S., Guo Y. (2019). Strengthening public health services to achieve universal health coverage in China. BMJ.

[27] Yong L. (2020). An empirical study on the selection behavior of patients in outpatient clinics under the background of hierarchical diagnosis and treatment. Chin. Hosp. Manag..

[28] National Bureau of Statistics (2017). China Health Statistical Yearbook 2017.

[29] Zen D., Li Z.X., Duan Z.Q., Liu D.P. (2015). Hospitalization options of patients in urban areas, Sichuan province. Mod. Prev. Med..

[30] Wensing M., Jung H.P., Mainz J., Olesen F., Grol R. (1998). A systematic review of the literature on patient priorities for general practice care. Part 1: Description of the research domain. Soc. Sci. Med..

[31] Kleij K.-S., Tangermann U., Amelung V.E., Krauth C. (2017). Patients’ preferences for primary health care—a systematic literature review of discrete choice experiments. BMC Health Serv. Res..

[32] Wang X., Jiang R., Li J., Chen J., Burström B., Burström K. (2018). What do patients care most about in China’s public hospitals? Interviews with patients in Jiangsu Province. BMC Health Serv. Res..

[33] Yip W.C.-M., Hsiao W.C., Chen W., Hu S., Ma J., Maynard A. (2012). Early appraisal of China’s huge and complex health-care reforms. Lancet.

[34] Barber S.L., Borowitz M., Bekedam H., Ma J. (2014). The hospital of the future in China: China’s reform of public hospitals and trends from industrialized countries. Health Policy Plan..

[35] Xiao Y., Qiu Q.M., Huang Y.X., Zhu S.Y. (2021). Patients gather in large hospitals: The current situation of chinese hospitals and the direction of medical reform. Postgrad. Med. J..

[36] Chen B., Li X.B., Lu Y.J. (2009). Agent-based modeling and simulation research into residents healthcare choice. Syst. Eng..

[37] Guo Y.T. (2014). Residents’ choice of medical services and its difference between urban and rural resident. Med. Philos..

[38] Wei M., Xiao J.C. (2014). Study on influencing factors and countermeasures analyses of choosing different medical institutions by patients. Chin. Health Serv. Manag..

[39] Centre for Health Statistics and Information, MOH China (2008). Analysis Report of National Health Services Survey in China.

[40] Wang M., Zhang K.J., Jiang L., Huang X., Bao S. (2010). Impact factors model of medical behavior of Chinese urban and rural residents. Chin. J. Gen. Pract..

[41] Lv L.H., Ji Y.D., Zhang Y.X., Li G.Z., Liu X.Q., Zhang B.L., Gao Z.Q., Tian H.Y. (2013). Demand for and intention to medical care among infertile men. Chin. J. Reprod. Health.

[42] Dou W.J., Zhao F., Gu J.L. (2015). Survey of rural residents’ wills to medical treatment and its influencing factors in Shandong province. Chin. J. Gen. Pract..

[43] Bing S.S., Yin A.T., Meng Q.Y. (2010). Study on the rural chronic patients’ medical treatment choice. Chin. Health Econ..

[44] Sun Z., Wang S., Zhao H., Yu H. (2020). Does Descending Resources Reform Improve Patient Satisfaction and Reshape Choice of Care Providers? A Cross-Sectional Study in Zhejiang, China. INQUIRY J. Health Care Organ. Provis. Financ..

[45] State Council (2021). Report on Government Work in 2021. http://www.china-cer.com.cn/guwen/2021021911402.html.

[46] Shen X., Yang W., Sun S. (2019). Analysis of the impact of china’s hierarchical medical system and online appointment diagnosis system on the sustainable development of public health: A case study of shanghai. Sustainability.

[47] Huang J., Fang S., Liang H., Liu S., Wang L., Lu W. (2018). Effect of the collaborative reform of family doctor contract service on residents’ health management. Chin. Health Resour..

[48] Huang J., Gao Z. (2017). Health management and effect analysis of family doctor system: A case study from changing district of Shanghai. China Health Insur..

[49] Huang J.L., Zhang T., Wang L., Guo D.F., Liu S.S., Lu W., Liang H., Zhang Y., Liu C. (2019). The effect of family doctor-contracted services on noncommunicable disease self-management in Shanghai, China. Int. J. Health Plan. Manag..

[50] Meng Q., Fang H., Liu X., Yuan B., Xu J. (2015). Consolidating the social health insurance schemes in China: Towards an equitable and efficient health system. Lancet.

[51] Lu C., Zhang Z., Lan X. (2019). Impact of China’s referral reform on the equity and spatial accessibility of healthcare resources: A case study of Beijing. Soc. Sci. Med..

[52] Li L., Fu H. (2017). China’s health care system reform: Progress and prospects. Int. J. Health Plan. Manag..

[53] Xie H.F., Zheng P.J., Ma J.W., Jiang J.H. (2020). Application of information system to the management of outpatient doctor scheduling. Chin. Hosp..

[54] Liu H. (2015). Does over treatment exist in China? Study on the efficiency of hospital care across provincial hospitals. Soc. Sci..

[55] Shin H.-W., Yoon J.-H., Noh Y.-H., Yeo J.-Y. (2014). The impact of supplier induced demand on increase in medical aid expenditure. Korean Acad. Health Policy Manag..

[56] Kuruppu J.C., Corretti M., Mackowiak P., Roghmann M.C. (2002). Overuse of transthoracic echocardiography in the diagnosis of native valve endocarditis. Arch. Intern. Med..

[57] Fabbrini G., Barbanti P., Pascali M.P., Lenzi G.L., Cerbo R. (1999). Impact of the International Headache Society criteria on the use of neuroimaging for headache diagnosis in a headache clinic. Headache.

[58] Jordan J.E., Ramirez G.F., Bradley W.G., Chen D.Y., Lightfoote J.B., Song A. (2000). Economic and outcomes assessment of magnetic resonance imaging in the evaluation of headache. J. Natl. Med. Assoc..

[59] Lester M.S., Liu B.P. (2013). Imaging in the evaluation of headache. Med. Clin. N. Am..

[60] Aykin T. (2000). A comparative evaluation of modeling approaches to the labor shift scheduling problem. Eur. J. Oper. Res..

[61] Sibbald B., Pickard S., McLeod H., Reeves D., Mead N., Gemmell I., Coast J., Roland M., Leese B. (2008). Moving specialist care into the community: An initial evaluation. J. Health Serv. Res. Policy.

[62] Bowling A., Bond M. (2001). A national evaluation of specialists’ clinics in primary care settings. Br. J. Gen. Pract..

